# qDESH: a method to quantify disproportionately enlarged subarachnoid space hydrocephalus

**DOI:** 10.1186/s12987-025-00677-2

**Published:** 2025-07-01

**Authors:** Sofia Behndig, Afroditi Lalou, Jan Axelsson, Jenny Larsson, Anders Wåhlin, Pavel Ryska, Ondrej Slezak, Katrine Riklund, Jan Zizka, Jan Malm, Anders Eklund

**Affiliations:** 1 Department of Diagnostics and Intervention, Biomedical Engineering and Radiation Physics, Umeå University, Umeå, Sweden; 2 Umeå Center for Functional Brain Imaging (UFBI), Umeå University, Umeå, Sweden; 3 Department of Clinical Sciences, Neurosciences, Umeå University, Umeå, Sweden; 4 Department of Applied Physics and Electronics, Umeå University, Umeå, Sweden; 5 Department of Diagnostic Radiology, University Hospital Hradec Kralove, Sokolska 581, 50005 Hradec Kralove, Czech Republic; 6 Department of Diagnostic Radiology, Faculty of Medicine in Hradec Kralove, Charles University in Prague and University Hospital Hradec Kralove, Sokolska 581, 50005 Hradec Kralove, Czech Republic; 7 Department of Diagnostics and Intervention, Diagnostic Radiology, Umeå University, Umeå, Sweden

## Abstract

**Background and purpose:**

Disproportionately enlarged subarachnoid space hydrocephalus (DESH) is a radiological biomarker for idiopathic normal pressure hydrocephalus (iNPH). DESH is a subjective measure, based on visual assessments, which may limit its reliability. The aim of this study was to develop and validate a method for the objective quantification of DESH.

**Materials and methods:**

By using a semiautomatic quantitative method, we calculated quantitative DESH (qDESH), defined as a ratio between CSF volumes at high convexities and Sylvian fissures. The analysis was based on three-dimensional T1-weighted images from 35 subjects with iNPH (mean age 74 yrs; 10 females) and 45 controls (mean age 72 yrs; 13 females). The interrater agreement for qDESH was evaluated by the intraclass correlation coefficient, and qDESH was compared with visual assessments performed by two neuroradiologists.

**Results:**

All subjects with iNPH and 13% of the controls visually scored DESH positive. The median qDESH was 2.48 (5th to 95th percentile 0.88 to 5.42) for iNPH and 0.63 (5th to 95th percentile 0.37 to 1.73) for the controls. The area under the receiver operating characteristic curve for qDESH was 0.95 (95% confidence interval 0.90–1) in separating iNPH patients from controls. The interrater agreement for qDESH was 0.99 (95% CI 0.986–0.994, *p* < 0.001).

**Conclusion:**

Unlike visual DESH, qDESH generates a continuous variable, enabling reproducible quantification of DESH severity. With this method we can objectively investigate the diagnostic accuracy and prognostic assessment of DESH in iNPH.

**Supplementary Information:**

The online version contains supplementary material available at 10.1186/s12987-025-00677-2.

## Introduction

Idiopathic normal pressure hydrocephalus (iNPH) is a syndrome of elderly, characterized by ventriculomegaly in combination with gait/balance disturbance with or without cognitive impairment. Symptoms of iNPH can be treated by insertion of a CSF shunt [1]. In addition to ventriculomegaly, imaging often reveals disproportion between sulcal effacement at high convexities and dilated Sylvian fissures [1, 2]. These structural changes have been named disproportionately enlarged subarachnoid space hydrocephalus (DESH) and are included in the Japanese iNPH guidelines and the iNPH Radscale [3, 4]. In iNPH research and patient care, the major challenges are identifying shunt responders and to exclude patients with other diagnoses. In radiological practice, DESH is commonly used to determine whether ventriculomegaly is caused by cerebral atrophy or hydrocephalus, and to identify iNPH patients who benefit from shunt placement [3, 4]. However, the prognostic impact of DESH remains unconfirmed with studies showing varying results [5, 6]. Two meta-analytic studies [7, 8] emphasize that no consensus has been reached on how to objectively and reproducibly evaluate DESH, with varying definitions and numbers of DESH grades being used. Although contradicting results exist, several studies have reported a positive predictive value of DESH [6, 9–12], indicating that DESH has the potential to become a prognostic marker of iNPH. Thus, to uncover the diagnostic and prognostic potential of DESH, more accurate methods to standardize DESH evaluation need to be developed.

Approximately one-fifth of the elderly population presents with ventriculomegaly on imaging [13], emphasizing the importance of correctly evaluating DESH. In routine radiological practice, this assessment relies on radiologists’ subjective visual judgment of a patient being DESH positive or negative, with considerable risk of low reproducibility. Quantitative methods of DESH assessment have previously been developed but they have not shown robust interrater agreement [2, 14–18]. To achieve consistency in DESH evaluation, a method of quantitative DESH assessment based on precisely defined anatomical landmarks still needs to be developed and validated. This also is an imperative for evidence-based evaluations of DESH in future studies.

The aim of this study was to create and validate a tool for quantitative grading of DESH severity. For this purpose, we introduce a semiautomatic segmentation tool based on precisely defined anatomical landmarks, generating a quantitative score named qDESH.

## Materials and methods

**Fig. 1:**
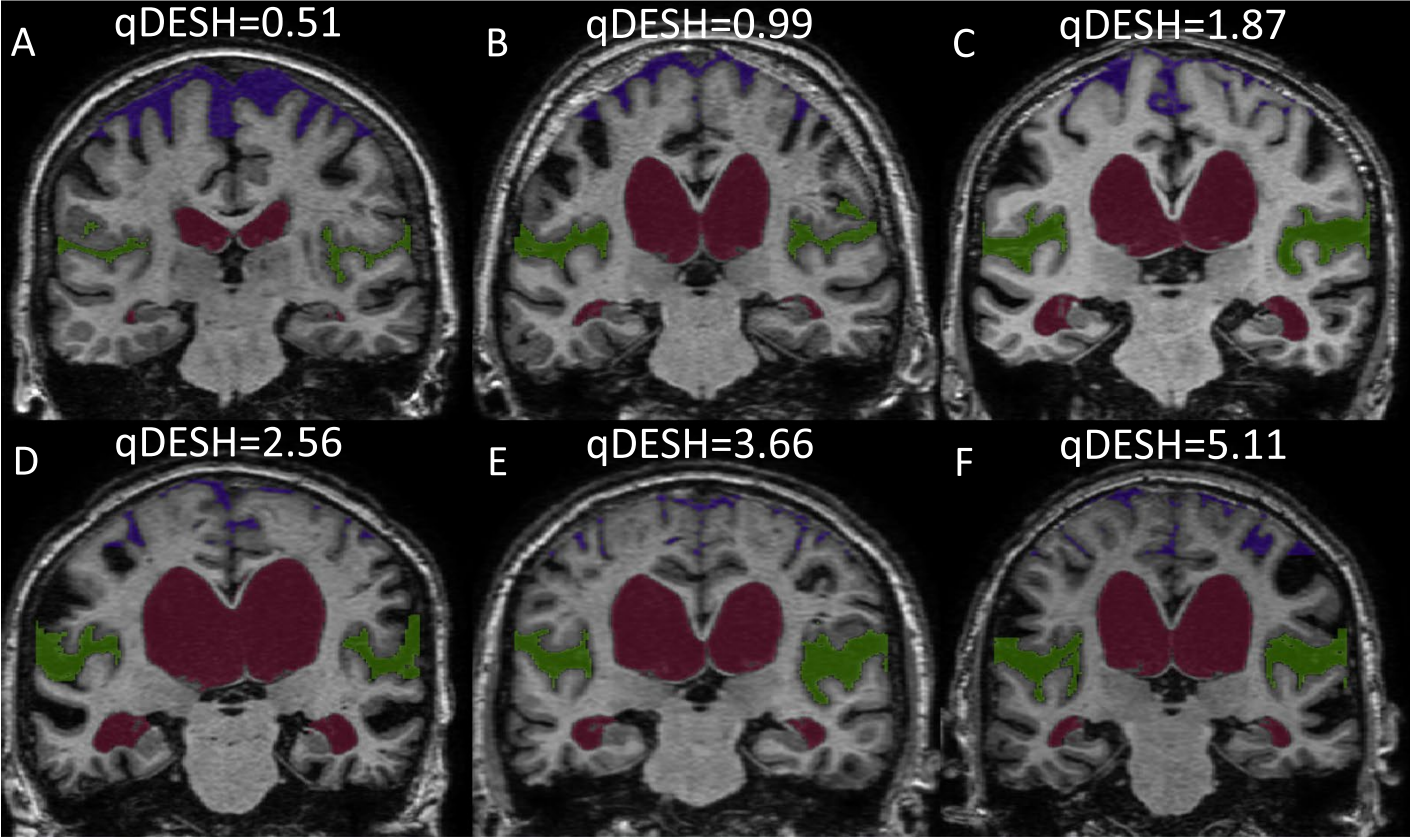
Six subjects with different qDESH viewed at the posterior commissure, one healthy control (**A**) and five subjects with iNPH (**B**–**F**). The images illustrate the CSF of the Sylvian fissures (green), high convexities (purple) and lateral ventricles (red). qDESH was calculated as a ratio between the CSF of the Sylvian fissures and high convexities

We present qDESH, an objective method for quantifying DESH on T1-weighted images using semiautomatic segmentation and manual delineation. By means of volumetric analysis of the ratio between anatomically well-defined CSF-volumes of the Sylvian fissures and high convexities (Fig. 1), a continuous quantitative measure of DESH (qDESH) is generated. The validity of qDESH was assessed by evaluating interrater reliability and comparing it to conventional visual assessment of DESH. Additionally, we compared qDESH between patients with iNPH and controls.

### Patients and controls

The study population consisted of 35 subjects with iNPH and 45 healthy controls. Both the iNPH and control group were included as part of previous studies [19–21].

The iNPH diagnosis was based on the American–European iNPH guidelines [22]. Thirty-one patients were classified as probable iNPH and four as possible iNPH. In total, thirty patients had a shunt surgery after they got their diagnosis.

Controls underwent examination by a neurologist, ruling out psychiatric, neurological, or advanced atherosclerotic diseases.

### Imaging parameters and preprocessing

For image analysis, 3D T1-weighted spoiled gradient echo sequences were acquired with MRI between 2007 and 2016. Four different scanners were used during this period (GE Healthcare Discovery MR750 3 T (n = 17), Philips Achieva 3 T (n = 60) and 1.5 T (n = 1), Achieva dStream 1.5 T (n = 2)), with slightly different scanning-parameters, resulting in voxel sizes between 0.53 and 0.95 mm^3^. The signal intensities of all MR images were bias corrected using SPM 12 [23]. All images were aligned with the anterior to posterior commissure line and mid-sagittal section, with the aid of the ‘acpcdetect’-program [24–26].

### Definition of qDESH

qDESH was calculated as the ratio between the CSF volumes of the Sylvian fissures (V_CSF-SF_) and high convexities (V_CSF-HC_) (*Eq. *1).

1 $$qDESH=\frac{{V}_{CSF-SF}}{{V}_{CSF-HC}}$$

**Fig. 2:**
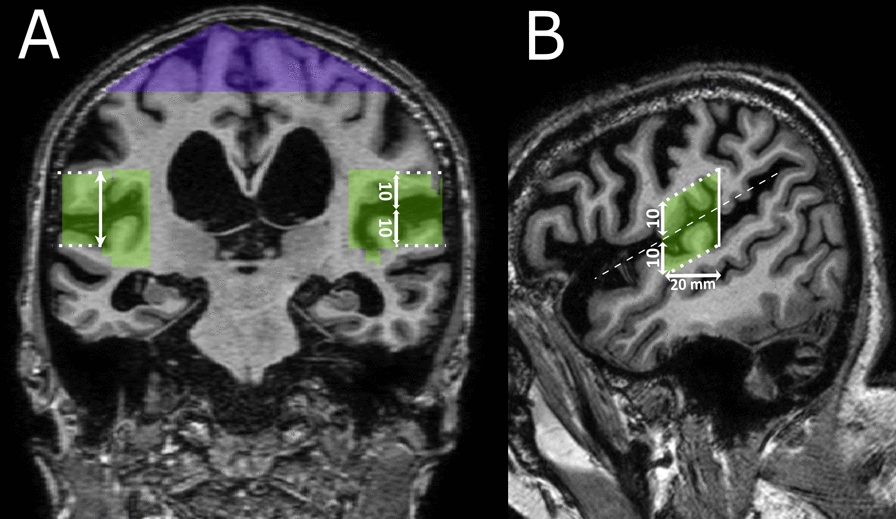
Search volume of the Sylvian fissures (green) and high convexities (purple). **A** Coronal slice at the posterior commissure that illustrates the opercular margin (dotted line), high convexities and Sylvian fissures search volumes. **B** Sagittal slice at the medial margin of the opercular margin (dotted line) showing the Sylvian fissure search volume spanning from the posterior commissure to 20 mm anterior to the posterior commissure (whole lines). The opercular margin was placed 10 mm superior and inferior (vertical arrows) from the center of the Sylvian fissure (dashed line)

The semiautomatic segmentation and manual delineation procedures are described on GitHub [27]. The definitions of V_CSF-SF_ and V_CSF-HC_ are illustrated in Fig. 2 and described as follows:

CSF was identified in two steps: first creating a volume (“search volume”), and second, performing a segmentation within this search volume. Separate search volumes were drawn over high convexities and Sylvian fissures with the dura mater as the outer limit (Fig. 2A). Considering previously published results [21, 28, 29] and to achieve precise anatomical definition, we restricted the search volume to the area between the posterior commissure and 20 mm anterior to the posterior commissure, perpendicular to the anterior to posterior commissure line (Fig. 2B). One more search volume was created over the lateral ventricle (described below). Within the search volumes, CSF volumes of interest were segmented using a global threshold defined as the signal intensity midway between the identified CSF and gray matter (Supplemental Fig. 1) inspired by the method of Kitagaki et al. [2].

**V**_**CSF-HC**_; we defined the high convexities search volume as the topmost cranial 30 cm^3^ of combined CSF and brain from both hemispheres (Fig. 2A). The segmented CSF within this search volume was denoted V_CSF-HC_.

**V**_**CSF-SF**_; the Sylvian fissures are defined as the region between the insula, frontoparietal, and temporal operculum [30]. To further delineate the Sylvian fissure, given its interindividual anatomical variability as well as highly variable perisylvian sulci, we included an additional margin around the operculum. As shown in Fig. 2, this margin was placed 10 mm superior and inferior to the center of the Sylvian fissures. The medial margin was aligned with the lateral-most convexity of the cerebellar hemisphere, as identified on sagittal slices (Fig. 2B). The segmented CSF-volume over both hemispheres was denoted as V_CSF-SF_.

### Segmentation and software

A step-by-step guide to the segmentation process and an instruction video can be found on GitHub [27]. The search volumes were created by two raters (S.B. and A.L.) using imlook4d [31], executed within MATLAB R2023b, Natick, Massachusetts: The MathWorks Inc.; 2022. The search volumes over the Sylvian fissures and high convexities were manually drawn. The search volume for the lateral ventricle was created using a flood-fill function. Final segmentation and qDESH analysis were performed utilizing the *deshify*-script (tag 1.0) [27] running in MATLAB. The *deshify*-script [27] depends on the Imlook4d-code [31] to run. Examples of extracted CSF-volumes for one slice are shown in Fig. 1.

### Categorization of visual DESH assessment by radiologists

The visual assessment of DESH was previously performed independently by two neuroradiologists (P.R. and O.S., 18 versus 8 years of experience) and was graded as *No* DESH (iNPH n = 0; controls n = 39), *Mild to moderate* DESH (iNPH n = 18; controls n = 5) or *Severe* DESH (iNPH n = 18; controls n = 1), with an intraclass correlation coefficient (ICC) of 0.89 [21]. The two neuroradiologists agreed in 81% of the cases. In this study, the higher grade was used in cases of neuroradiologists’ disagreement.

### Linear measures

The same two neuroradiologists who performed the visual DESH assessment also measured Evans index, z-Evans index, callosal angle and brain to ventricle ratio (BVR) [21]. Their measurements angulated to the anterior to posterior commissure plane are reported in this study.

### Statistics and data analysis

*P*-value of < 0.05 was considered significant. Statistical analyses were performed using IBM SPSS Statistics (Version 27) and R (version 4.3.2). The values (qDESH, V_CSF-SF_, V_CSF-HC_) of the individual raters (S.B. and A.L.) were combined as a mean unless stated otherwise.

For validation, qDESH was compared to the visual DESH grades using nonparametric tests (Kruskal‒Wallis test and Mann‒Whitney U test) as qDESH was not normally distributed (Shapiro‒Wilk test, *p* < 0.001).

Discriminative power between iNPH patients and controls was evaluated separately for qDESH, V_CSF-SF_, V_CSF-HC_, and lateral ventricles using the area under the curve (AUC) from receiver operating characteristic (ROC)‒curves. Differences between the AUCs were tested using the DeLong-test for correlated ROC‒curves.

To evaluate possible relationships between qDESH, V_CSF-SF,_ and V_CSF-HC_ to lateral ventricular volume and age, correlations were done using Pearson’s and Spearman’s correlations.

Previous research using visual assessment had often been based on an unspecified slice position. Therefore, we decided to assess whether DESH evaluation differed depending on the slice position. For this purpose, a separate qDESH (qDESH_slice_) was calculated for each individual coronal section.

Interrater agreement between the two raters was analyzed for qDESH, V_CSF-SF_, V_CSF-HC_, and lateral ventricles using ICC with a two-way mixed model, absolute agreement, and single measures. qDESH differences between the raters were visualized using a Bland–Altman and scatter plot. The dependency of the raters’ difference to their average qDESH-score was assessed using linear regression.

Differences between groups were tested using independent samples and difference between raters with a paired t-tests.

## Results

### qDESH versus visual assessment of DESH

**Fig. 3:**
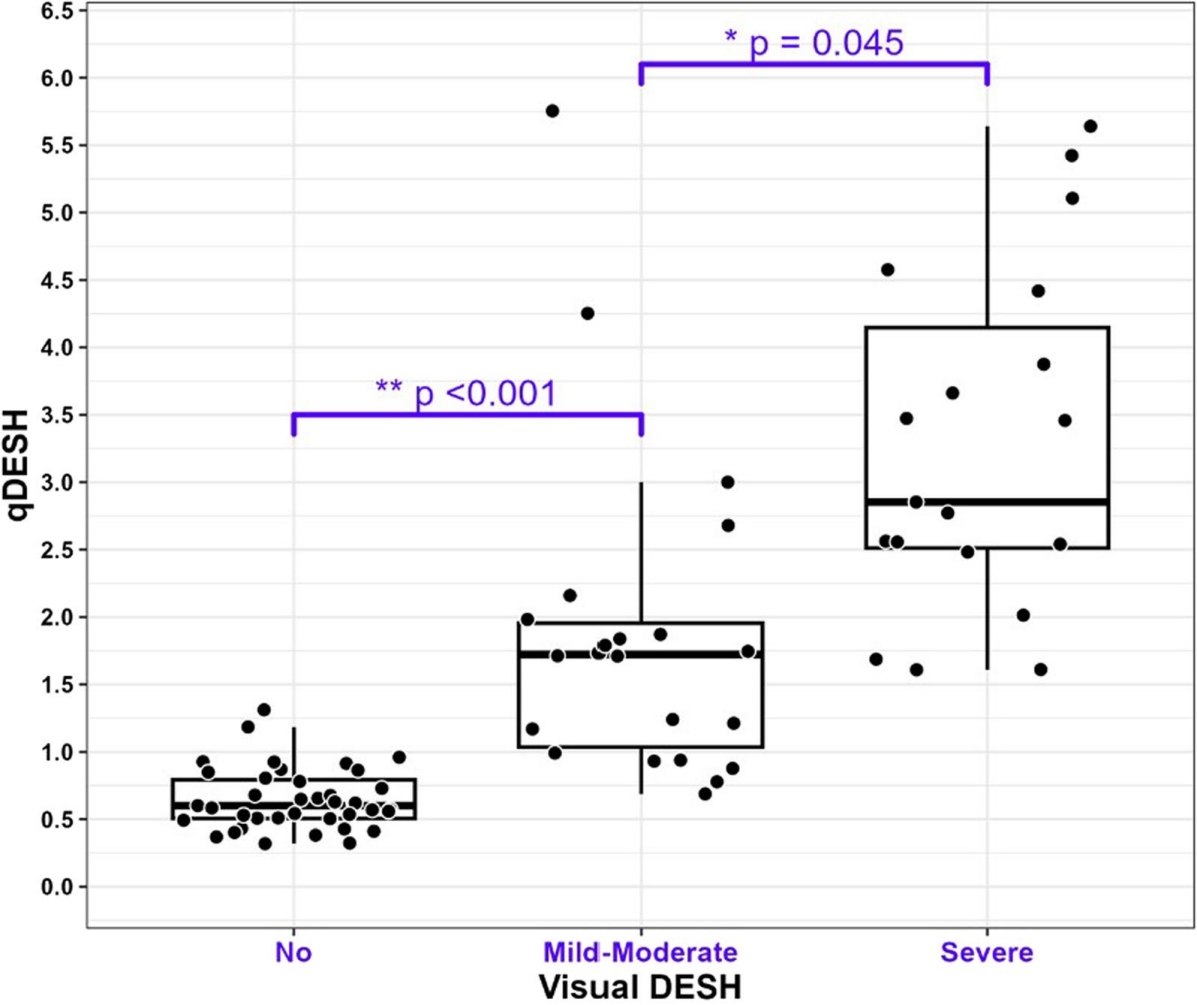
Box plot of every subject’s qDESH categorized to its corresponding visual disproportionately enlarged subarachnoid space hydrocephalus (DESH) assessment grades

Demographics are summarized in Table 1. Median qDESH for all subjects was 0.93 (range 0.32–5.75). As shown in Fig. 3, qDESH identified DESH as intended, i.e. the qDESH-score differed between the groups *No*, *Mild to moderate*, and *Severe* DESH (Kruskal Wallis test; *p* < 0.001). Thus, qDESH was in accordance with the traditional visual grading performed by two experienced neuroradiologists.

**Table 1:** Demographics

	iNPH (n = 35)	Controls (n = 45)
Age, y ± SD	74 ± 7.2	72.6 ± 5.7
F/M	10/25	13/32
qDESH, median (5–95 percentile)	2.48 (0.88–5.42)	0.63 (0.37–1.73)
Evans index	0.38 ± 0.04	0.29 ± 0.03
z-Evans index	0.42 ± 0.04	0.28 ± 0.03
Callosal angle	69.9 ± 21.4	115.6 ± 13.9
BVR	0.93 ± 0.2	2.54 ± 0.85

*iNPH* idiopathic normal pressure hydrocephalus, *SD* Standard deviation, *BVR* Brain to ventricle ratio

### Differentiation between iNPH patients and controls

In the ROC-analysis, a cutoff value of qDESH = 1.0 yielded a sensitivity of 94% and a specificity of 89%. Compared with the parameters in the qDESH formula (Eq. 1), qDESH showed a greater AUC (0.95, 95% CI 0.90–1) than V_CSF-SF_ (0.89, 95% CI 0.81–0.97) and V_CSF-HC_ (0.91, 95% CI 0.85–0.98), however, the difference was not significant.

Considering that six subjects in the control group were DESH positive on visual scoring and might therefore not be seen as suitable controls to identify a qDESH cutoff value, a separate analysis was performed excluding these subjects. The resulting cutoff value found by the ROC-analysis remained at qDESH = 1.0, with a sensitivity of 94% and specificity of 95%. The six subjects excluded for this sub analysis did not differ from the remaining part of the control group with regards to Evans index, callosal angle, BVR or qDESH. Z-Evans index was slightly higher but did not reach the values of the iNPH-group.

### qDESH in iNPH

**Fig. 4:**
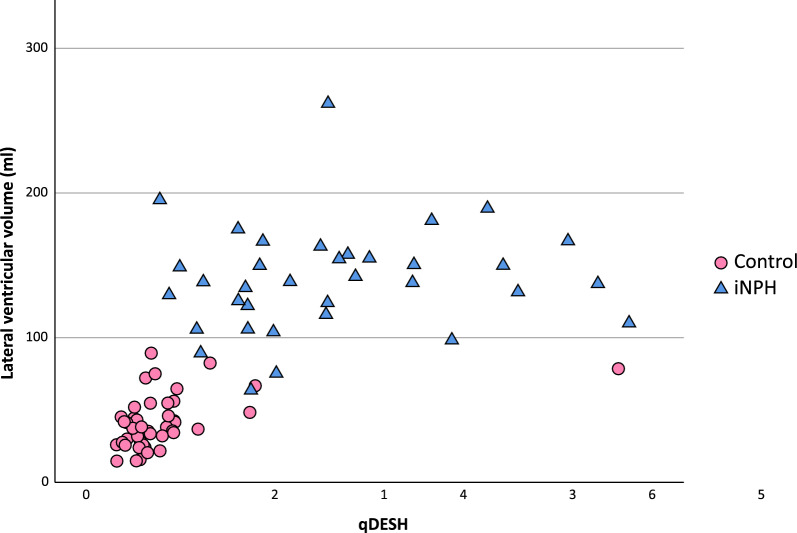
Scatterplot with the linear regression between qDESH and the lateral ventricular volume. The overall correlation for both groups was R = 0.642 (p < 0.001). The corresponding value for iNPH (blue triangle) was 0.123 (Pearson, p = 0.483) and for healthy controls (pink circle) 0.469 (Spearman, p = 0.002). The control with qDESH > 5 had several imaging features of iNPH but was confirmed clinically healthy at the time of investigation. *iNPH *idiopathic normal pressure hydrocephalus

All subjects in the iNPH group showed DESH positive on visual scoring, either of *Mild to moderate* (n = 17, median qDESH = 1.74) or *Severe* degree (n = 18, median qDESH 2.81, *p* = 0.007). The qDESH 5th to 95th percentile range was 0.88–5.42 (median 2.48). There was no correlation between qDESH, or its separate components (V_CSF-SF_ and V_CSF-HC_), and the lateral ventricular volume (Fig. 4*, *Table 2). Likewise, there was no correlation with age* (*Table 2). When exploring the correlation between qDESH and linear ventricular measures further, there was no significant correlation (Pearson) to Evans index (R = 0.01, *p* = 0.57), z-Evans index (R = 0.29, *p* = 0.08), callosal angle (R = − 0.27, *p* = 0.12) or BVR (R = − 0.25, *p* = 0.14).

### qDESH in the control group

In the control group, 87% (n = 39) were visually graded as *No* DESH, five as *Mild to moderate* DESH and one as *Severe* DESH, who also had a high qDESH-score (5.64) (Fig. 4). The qDESH in the 5th to 95th percentile range in the control group was 0.37 to 1.73 (median 0.63). There was a correlation between lateral ventricular volume and qDESH (Spearman’s R = 0.47, *p* = 0.001) (Fig. 4, Table 2), and between age and V_CSF-SF_ (Table 2).

**Table 2:** Correlations between the lateral ventricular volume and age to qDESH, V_CSF-SF_ and V_CSF-HC_

	Lateral ventricular volume		Age	
	iNPH	Controls	iNPH	Controls
qDESH	0.123_a_	0.469**_b_	0.065_b_	0.176_b_
V_CSF-SF_	− 0.09_a_	0.377*_b_	0.026_a_	0.346*_b_
V_CSF-HC_	− 0.247_a_	− 0.387**_a_	0.086_a_	0.034_a_
Lateral ventricular volume	N/A	N/A	− 0.049_a_	0.230_b_

^*^ = p < 0.05, ** = p < 0.001. _a_ = Pearson’s correlation, _b_ = Spearman’s correlation

*CSF* Cerebrospinal Fluid, *V *_*CSF-SF*_CSF volume of the Sylvian fissures, *V*_*CSF-HC* _CSF volumes of the high convexities, *iNPH* idiopathic normal pressure hydrocephalus

### Interrater agreement

**Fig. 5:**
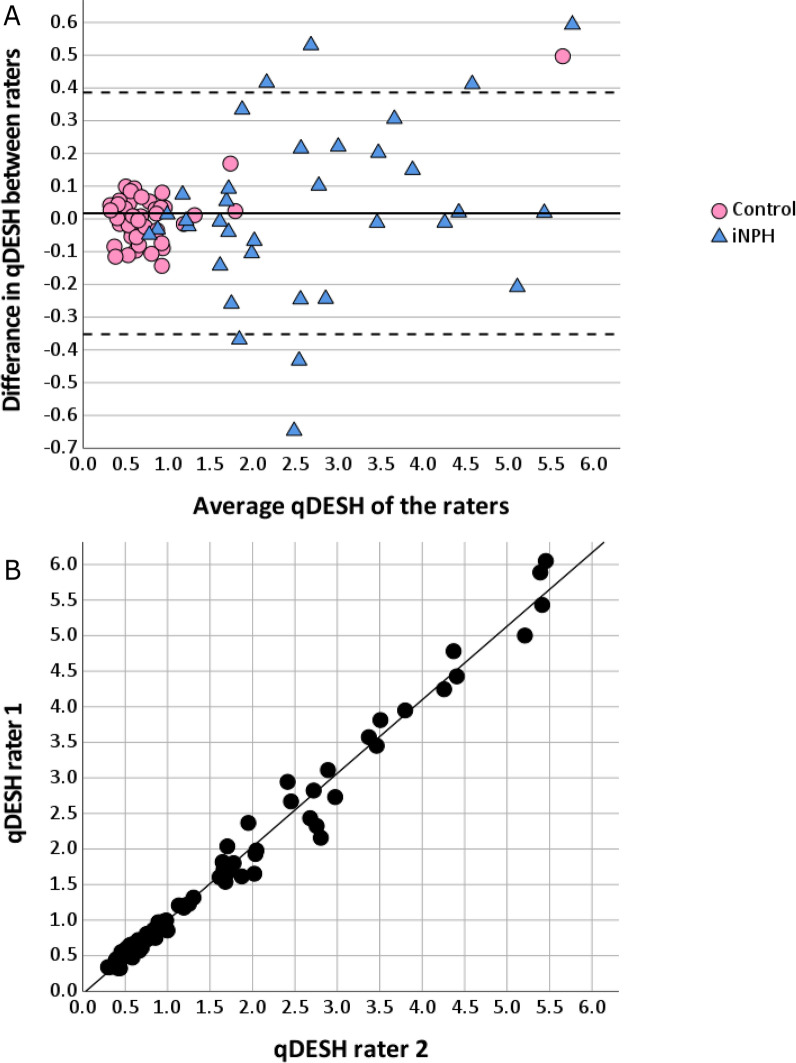
**A** Bland-Altman plot illustrating the difference in qDESH between the raters. The solid line represents the mean difference, and the dashed lines the 95% confidence interval. Subjects with idiopathic normal pressure hydrocephalus are displayed as blue triangles and controls in pink circles. The control with qDESH > 5 had several imaging features of iNPH but was confirmed clinically healthy at the time of investigation. **B** Scatter plot of the two raters’ qDESH-scores with regression line

When comparing the agreement between the two independent raters in delineating the CSF-volumes and qDESH, the resulting ICCs were: qDESH 0.99 (95% CI 0.986–0.994, *p* < 0.001); V_CSF-SF_ 0.95 (95% CI 0.928–0.970, *p* < 0.001); V_CSF-HC_ 0.99 (95% CI 0.988–0.995, *p* < 0.001). The qDESH Bland‒Altman plot (Fig. 5A) demonstrated no mean difference between the raters (0.017, *p* = 0.4; Fig. 5A, B).

### Slice selection variability

**Fig. 6:**
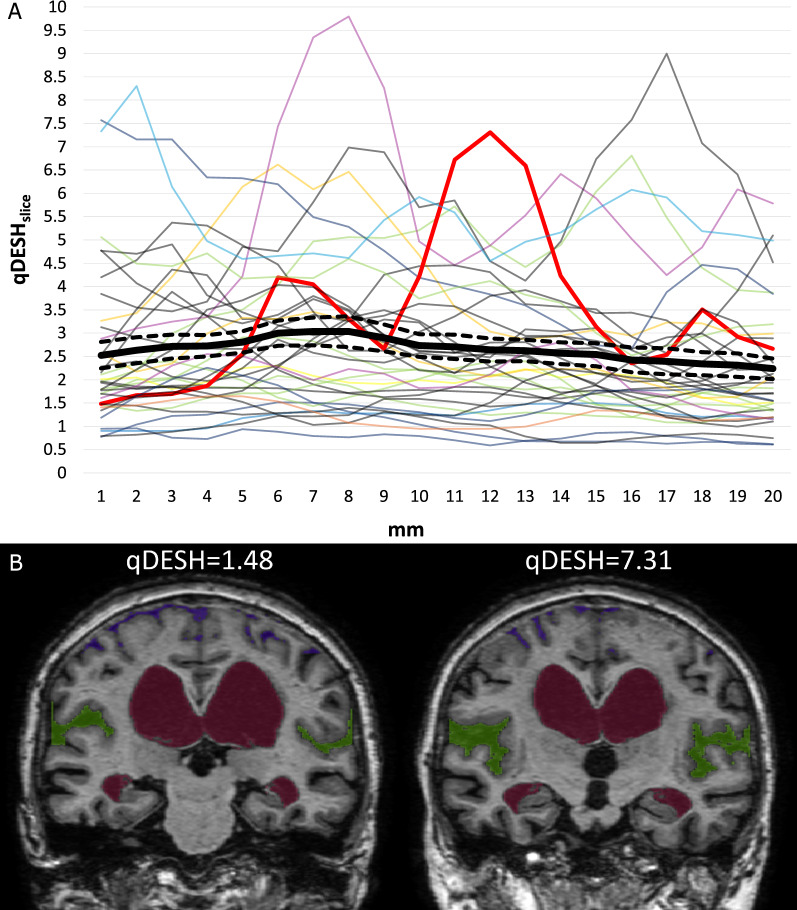
**A** Variation in qDESH per every coronal mm, in all subjects diagnosed with idiopathic normal pressure hydrocephalus (iNPH). The image illustrates the mean qDESH_slice_ for all subjects per coronal mm (solid black line), starting from the posterior commissure, with its standard error of the mean (dashed black line). The red line highlights the variation in one subject. The highest and lowest qDESH_slice_ for this patient are displayed in B. **B** Coronal slice of the lowest and highest qDESH_slice_ in the red-lined subject from A. The qDESH-score of this subject was 3.0

Previous research using visual assessment had often been based on an unspecified slice position. To evaluate intraindividual variability in DESH depending on the selected slice, qDESH_slice_ was determined for all slices. There was substantial variation (mean standard deviation = 0.68) in the iNPH group (Fig. 6A, exemplified in Fig. 6).

There was a seemingly random pattern in qDESH_slice_ with respect to the slice position (Fig. 6A).

## Discussion

This study aimed to develop a novel method to quantitatively measure DESH (termed qDESH). This method has proven valid and reproducible, providing an objective and quantitative measure of DESH on a continuous scale. qDESH is useful in clinical research where visual assessment of DESH has been an important but subjective parameter. As an objective measure, qDESH can be used to describe patient cohorts and to determine the diagnostic and prognostic relevance of DESH in iNPH.

When reviewing the definition and reproducibility of DESH in the iNPH radiological literature, the need to develop methods that objectively quantify DESH becomes evident. Neuroimaging primarily aims to confirm the iNPH diagnosis and exclude differential diagnoses, differentiating atrophy-related ventriculomegaly from treatable hydrocephalus. With the introduction of CT in the 1970s, high convexity tightness was suggested for this purpose [32, 33]. Later studies proposed Sylvian fissure dilation as a feature of iNPH [2, 34]. Thus, ventriculomegaly combined with high convexity tightness and Sylvian fissure dilation was introduced as an MRI and CT biomarker of iNPH [34]. Although early assessments of DESH were based on voxel-based morphometry [2], most current studies rely on subjective visual inspection with varying DESH grades [7, 8]. To standardize DESH analysis and enable improved clinical care and future evidence-based research, we developed a tool allowing robust quantification of iNPH-related features of DESH.

The overall performance of qDESH in this study, as indicated by a high area under the ROC-curve, confirms that qDESH can distinguish healthy elderly from iNPH. Additionally, qDESH shows reliable agreement with visual assessment of DESH as evaluated by experienced neuroradiologists (Fig. 3). This confirms that the qDESH method can identify DESH as intended and is not measuring any other entity. We also note that the 95th percentile qDESH in healthy elderly was 1.73, providing a cutoff value of what could be considered a normal qDESH.

This study suggests that iNPH research may benefit from using qDESH because of its high reproducibility (ICC of 0.99). We have not found any previously developed method to quantify DESH that had robust interrater agreement [2, 14–18]. Previous research with visual DESH assessment reported interrater agreements (both ICC and Cohen ĸ) between 0.36 and 0.89 [4, 6, 10, 21]. Possible explanations for this inconsistency include variability in physicians’ clinical experience and different definitions and interpretations of DESH. As shown in Fig. 6A and B, qDESH_slice_ can vary considerably across the series of coronal slices within the same brain, e.g. differing between 1.48 and 7.31 in a single iNPH subject. This indicates that DESH assessments, both visual and quantitative, can vary substantially depending on the slice position, which typically is not specified in previous research [7, 8]. Virhammar et al. demonstrated that Sylvian fissure sizes can visually vary within a subject simply by manipulating the imaging angle [10]. Both this article and previous research thus promote standardization of DESH assessment, preferably by using a multislice or volumetric approach to enable proper evaluation of its clinical importance.

The continuous scale of qDESH could further contribute by providing better detail to the degree of disproportion between V_CSF-SF_ and V_CSF-HC_ than visual assessment could. Even though all iNPH subjects in our cohort were visually classified as DESH positive, there was a large spread in qDESH, covering most of the total qDESH range (Fig. 4). This study shows that quantifying DESH is not as simple as negative or positive grading [4–6, 9, 10] suggesting that qDESH may open for improved diagnostic and prognostic comparisons among “DESH-positive” subjects.

The continuous scale of qDESH enables objective comparison with other parameters. For iNPH subjects, we found no correlation between qDESH and lateral ventricular volume, linear measures or age. qDESH thus seems to assess a separate structural feature compared with routinely used iNPH radiological biomarkers. Ventriculomegaly in iNPH has been hypothesized to have an upward predilection, resulting in compression of CSF-spaces at high convexities [18, 35], which would lead to increased subarachnoid space disproportion (larger qDESH). A related theory is that tight convexities can obstruct the glymphatic pathway in this region. This is exemplified by studies with intrathecal gadolinium and repeated MRI showing that contrast enhancement at the high convexities is delayed in iNPH [36]. The availability of a quantitative measure of DESH can thus be used in glymphatic research of iNPH. Although we observed no relationship between ventriculomegaly and qDESH in iNPH patients in this study, the theories above could explain the difference between the iNPH and control groups (Fig. 4). It is also intriguing to hypothesize that subjects with ventriculomegaly and lower qDESH have more atrophy than those with ventriculomegaly and higher qDESH, but this requires further research to answer.

At this stage, qDESH is meant to assist in clinical research, with manual delineation and segmentation taking approximately 10–15 min. qDESH can be used to investigate whether the degree of DESH has any clinically relevant meaning for diagnostic and prognostic decisions. Our findings motivate future studies on clinical relevance, in a cohort of subjects investigated for suspected iNPH. If qDESH proves useful for diagnosis and prognosis in iNPH, efforts to automate the process can be made to enhance its clinical utility. To simplify future automation, we have created precise definitions of anatomical landmarks. The Sylvian fissures are defined in the literature [30]. However, no previous quantitative DESH method has considered perisylvian sulci. To avoid subjective decisions of when to include perisylvian sulci in the Sylvian fissure search volumes to the rater, we have defined the opercular margin (Fig. 2). The high convexities are less well-defined. Yamada et al. [16] recently published a definition of the high convexities, covering a larger area around the midline. However, their segmentation method is limited, as they discuss themselves, since it is not validated and requires anatomical expertise. To achieve high reproducibility of V_CSF-HC_ with minimal influence of focally enlarged sulci, we have proposed a consistent 30 cm^3^ search volume.

Different artificial intelligence (AI) methods are likely to play a major role in the segmentation and classification of radiological images in the future. In the setting of DESH this has recently been suggested by Yamada et al. [37], who utilized machine learning to perform automatic segmentation. AI methods have the advantages of obtaining fast results but can be limited in explainability. In this study we choose the path of developing qDESH based on well-defined anatomical landmarks, both to enhance explainability and the pathophysiological understanding of iNPH. A well-defined explainable segmentation step is important for proper investigation of the clinical relevance of DESH. In the future, if qDESH proves to be clinically relevant, the logical next step is to develop AI-based segmentation for faster qDESH calculations.

## Limitations and future development

We did not exclude focally enlarged sulci from V_CSF-HC_. Only four subjects displayed focally enlarged sulci in their V_CSF-HC_, none having a normal qDESH. Even though not shown relevant here, it may be worthwhile to develop a robust method to exclude focally enlarged sulci. The difference in resolution between subjects (both groups) could influence CSF segmentation, with lower resolution giving falsely low volumes in otherwise tight spaces (V_CSF-HC_). Thus, for patients with tight high convexities (low V_CSF-HC_), lower resolution could overestimate qDESH.

## Conclusions

qDESH facilitates objective and reproducible quantification of DESH severity, thus surpassing binary categorizations of DESH as simply positive or negative. This approach can be used when further exploring possible associations between DESH and iNPH pathophysiology.

## Supplementary Information

Additional file 1: Supplemental figure 1: Histogram over the signal intensity for the voxels within all search volumes (Sylvian fissure, high convexities and lateral ventricle). A global threshold (black line) is calculated as the signal intensity midway between identified cerebrospinal fluid (CSF) (blue line) and grey matter (red line). The green curve represents a moving-average filtered data used for identification of correct peak positions

## Data Availability

The datasets analyzed during the current study are available from the corresponding author on reasonable request.

## Abbreviations

iNPH: Idiopathic normal pressure hydrocephalus
DESH: Disproportionately enlarged subarachnoid space hydrocephalus
V_CSF-SF_: CSF volume of the Sylvian fissures
V_CSF-HC_: CSF volume of the high convexities
ICC: Intraclass correlation coefficient
AUC: Area under the curve
ROC: Receiver operating characteristics
