# Supplementary figures and images for: qDESH: a method to quantify disproportionately enlarged subarachnoid space hydrocephalus
Source: Fluids Barriers CNS. 2025 Jul 1;22:67. doi: 10.1186/s12987-025-00677-2 (PMC12219777; doi:10.1186/s12987-025-00677-2)

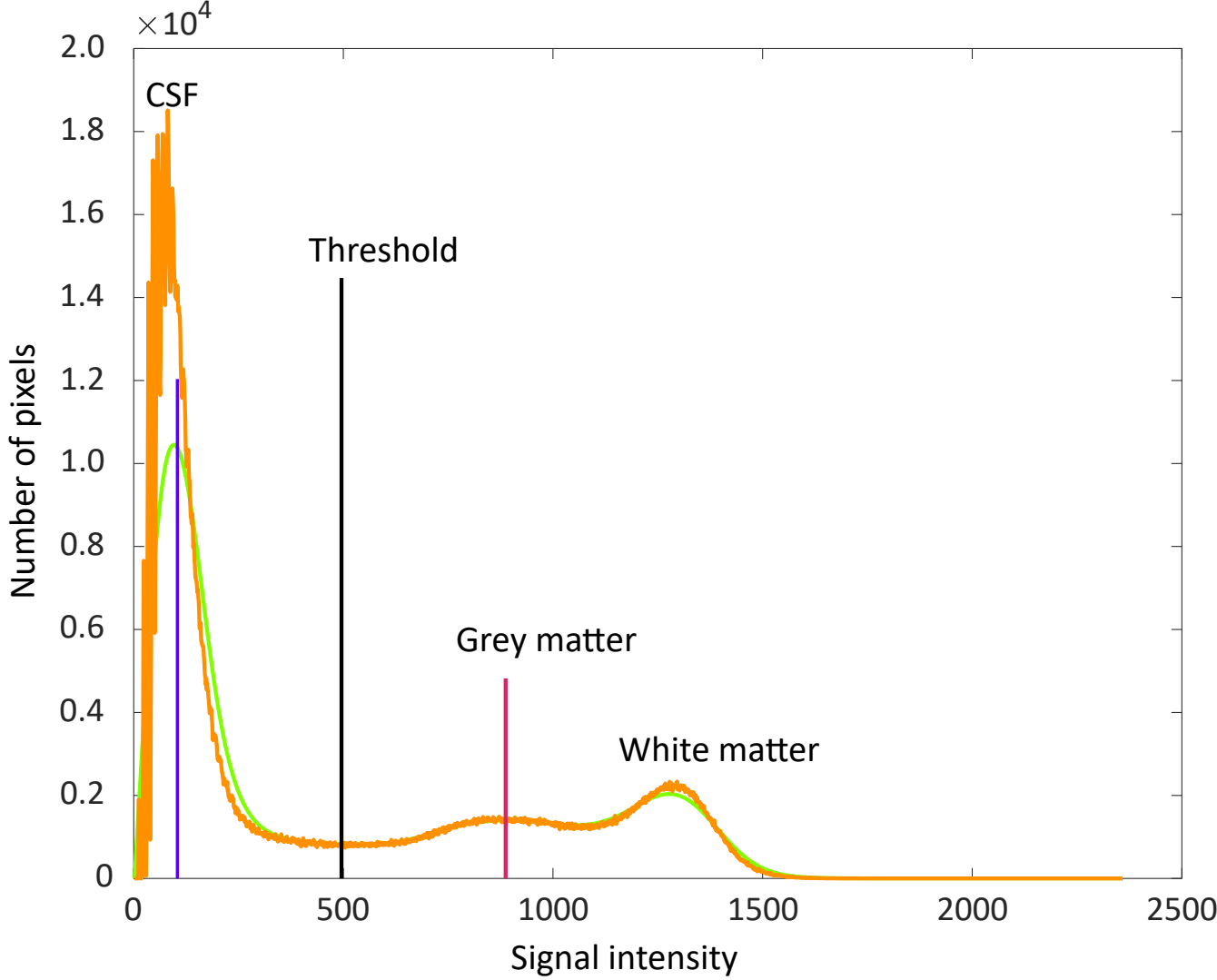

Supplement: Supplementary file 1 — Additional file 1: Supplemental figure 1: Histogram over the signal intensity for the voxels within all search volumes (Sylvian fissure, high convexities and lateral ventricle). A global threshold (black line) is calculated as the signal intensity midway between identified cerebrospinal fluid (CSF) (blue line) and grey matter (red line). The green curve represents a moving-average filtered data used for identification of correct peak positions [file 12987_2025_677_MOESM1_ESM.pdf]
